# Caspases as prognostic markers and mortality predictors in acute organophosphorus poisoning

**DOI:** 10.1186/s43141-020-00024-y

**Published:** 2020-04-13

**Authors:** Shimaa Tallat, Rania Hussien, Rania Hassan Mohamed, Mahmoud B. Abd El Wahab, Magdy Mahmoud

**Affiliations:** 1 Poison Control Center–Ain Shams University Hospitals (PCC-ASU), Cairo, Egypt; 2 Forensic Medicine and Clinical Toxicology Department, Faculty of Medicine, Ain Shams University, Cairo, Egypt; 3 Department of Biochemistry, Faculty of Science, Ain Shams University, Cairo, Egypt

**Keywords:** Acute organophosphorus poisoning, Apoptosis, Oxidative stress, Caspases, Genotoxicity

## Abstract

**Background:**

Organophosphorus (OP) compounds have been widely available for decades in agriculture for crop protection and as cheap pest controllers, which increases the rate of exposure and poisoning cases. Using serum cholinesterase as prognostic markers for the acute OP toxicity is controversial; therefore, we aim to find out prognostic biomarkers that best correlate with mortality and outcomes of patients with acute OP toxicity. Levels of serum oxidative stress biomarkers (malondialdehyde (MDA) and total antioxidant capacity (TAC)) and activity of the apoptotic biomarkers (caspase 3 and caspase 9) and pseudo-cholinesterase (p.ChE) were performed. Also, we evaluated the apoptotic capacity through determining the genotoxic effects and chromosomal abnormalities among OP intoxicated patients.

**Results:**

We found the activity of caspases and serum MDA and TAC were significantly increased after OP poisoning and decreased after the appropriate atropine and oxime treatment course. The ROC curve suggested caspases as mortality and outcome predictive markers for acute OP poisoning patients. However, OP poisoning cases before treatment showed significant DNA damage, and they did not show any chromosomal aberration.

**Conclusion:**

The mentioned results strongly suggest apoptotic-related markers (caspase 3, caspase 9) as prognostic markers for evaluation of the treatment, outcomes, and mortality rate in the acute OP toxicity patients.

## Background

OP compounds have largely been used as easy available and cheap pesticides worldwide. Ingestion of OP compounds for committing suicide is a major problem, especially for the developing countries, while accidental exposure is the most common cause of mild poisoning [1]. According to the World Health Organization (WHO), 3 × 10^6^ cases of pesticide poisoning (mainly OP compounds) occur every year, resulting in an excess of 250,000 deaths [2]. During 2017, PCC-ASUHs recorded 2290 acute OP poisoning cases, which represented nearly 10% of the total admitted patients during this year. In addition, OP poisoning deaths in the same year represented nearly 26% of the total mortality [3].

Acute OP toxicity occurs through inhibition of acetylcholinesterase (AChE) activity, resulting in accumulation of acetylcholine at the nerve endings, which attacks the brain and the nervous system. There are 2 different types of cholinesterase in the human body, which differ according to their tissue distribution and physiological functions. These two types are acetylcholinesterase (true cholinesterase, AChE), which occurs in higher concentration in central nervous system and the outer membrane of red blood cells, in addition to the cholinesterase present in plasma, liver, cerebrospinal fluid, and glial cells (pseudo-cholinesterase, p.ChE) [4]. The ratio of p.ChE to AChE in human plasma is 1000 to 1 [5]. During exposure to OP compounds, decrease in p.ChE activity usually falls first, followed by the decrease in red blood cell AChE activity. The p.ChE activity usually recovers before red blood cell AChE activity, returning to normal within a few days after a mild exposure in the absence of a repeated exposure [6]. Symptoms of toxicity include sweating, vomiting, diarrhea, pinpoint pupil, pulmonary crepitation, salivation, and muscle weakness. In severe poisoning cases, OP compounds cause coma, respiratory muscle failure, and death [7]. OP compounds also induce oxidative stress leading to generation of free radicals, increase of lipid peroxidation (LPO), and reduction of glutathione level. LPO is a destructive self-repetition chain reaction releasing MDA as its end product [8, 9]. This oxidative stress causes DNA damage, chromosomal aberration, and activation of apoptosis-related markers p53, caspase 3, and 9 [10, 11]. Both clinical features and laboratory parameters are very important for accurate diagnosis of cholinesterase inhibitor (ChEI) poisoning and detection of its severity [12]. In acute ChEI insecticides poisoning (e.g., OP compounds), low serum cholinesterase level supports the diagnosis of poisoning, but its significance for severity and mortality evaluation has been controversial [13, 14]. Therefore, there is a need for new quantifiable biochemical markers to the mortality prediction and prognosis of acute OP toxicity [13]. In our study, we aim to find out the prognostic biomarkers and to identify their cutoff values that best correlate with mortality of patients with acute OP toxicity. We assessed the oxidative stress parameters (TAC, MDA) and apoptotic-related markers (caspase 3, caspase 9) in patients with OP poisoning before and after treatment. In addition, we examined the genotoxic effects and chromosomal abnormalities among the intoxicated patient.

## Methods

This study included thirty OP intoxicated cases of both sex in the age of 18–55 years, who admitted to the PCC-ASUH and ten healthy subjects as control. The diagnosis of OP intoxication was based on the criteria guided by [15]. All included patients were treated on admission with obidoxime of 500 mg by slow intravenous injection and loading doses of atropine till dryness of chest secretions. Patients with age under 18 years, previous treatment of OP poisoning in another institutions, as well as patients with previous renal, hepatic, cardiovascular, or respiratory diseases, were excluded. Ethical committee approval was obtained, and informed written consents were signed by participants or their guardians to be included in this study. The demographic data including age, sex, delay time, and manner of poisoning were collected. In addition to clinical data such as length of hospital stay, the need for mechanical ventilation, and the outcome were also recorded.

### Samples

Serum samples were collected under aseptic conditions twice from patients on admission before starting the treatment (atropine and oximes) and within 1 week of treatment before discharge. Blood samples were collected on heparinized vacutainer tube, on admission before starting the treatment, for Comet assay and karyotyping. Informed written consents were signed by participants or their guardians to be included in this study.

### Gas chromatography

OP residue analysis of the collected samples was carried out in three steps: extraction of OP residues from serum samples, cleaning, and type determination by gas chromatography according to [16]**.** Briefly, all the extracted and cleaned-up samples were kept at – 20 ^°^C. Qualitative analysis of chlorpyrifos, azinophos ethyl, propenfos, dichlorovos, methamidophos, ethoprophos, chlorpyrifos-methyl, dimethoate, phenothoate, prothiphos, ethion, triazophos, and malathion residues were performed by using gas chromatograph (GC) and Hp 6890 serial equipped with flame photometric detector (FPD) operated in the phosphorus mode (529-nm filter). The purified samples were dissolved in a known volume of ethyl acetate and injected under the following conditions: capillary column DB-5MS (30 m × 0.32 mm i.d. × 0.25-μm film thickness), detector temperature was 250 °C, injector temp was 250 °C, and the column temperature was programmed at 160 °C, hold for 2 min, then raised to 240 °C at a rate of 6 °C/min., and hold for 5 min. Nitrogen carrier gas flow was 3 ml/min, and hydrogen flow was 200 ml/min.

### Serum biochemical analysis

Serum p.CHE activity has been measured by the kinetic method according to [17]. The serum cholinesterase catalyzes the hydrolysis of the butyryl-thiocholine (BTC), forming butyrate and thiocholine. The thiocholine reduces the exacyanoferrate (III) to exacyanoferrate (II), whose absorbance is directly proportional to p.CHE activity.

Serum total antioxidant capacity (TAC) was measured by the colorimetric method according to [18]. The antioxidants in the sample eliminate a defined amount of exogenously provided hydrogen peroxide (H_2_O_2_). The residual H_2_O_2_ is determined calorimetrically by an enzymatic reaction, which involves the conversion of 3,5 dichloro-2-hydroxybenzensulphonate to a colored product.

Serum malondialdehyde was measured by the colorimetric method according [19]. Thiobarbituric acid (TBA) reacts with malondialdehyde (MDA) in acid medium at 95 °C for 30 min to form thiobarbituric acid reactive product. The absorbance of the resultant pink product can be measured at 534 nm.

Serum apoptosis-related genes caspase 3 and 9 activity was measured by enzyme-linked immunosorbent assay (ELISA) according to the manufacturer’s instructions (BlueGene Biotech®; Shanghai, China). Briefly, serum samples were added to the wells pre-coated with caspase 3 or 9 monoclonal antibody. After incubation, a biotin-conjugated anti-human caspase 3 or 9 antibodies were added. Then, streptavidin-HRP was added followed by the substrate solution, and the colored product was measured at 450 nm.

### Genetic analysis

#### DNA damage studies

It was carried out using the comet assay, single cell gel electrophoresis (SCGE) as described by [20] with slight modifications. Ninety microliters of 1% low melting agarose and 10 μl of sample (heparinized blood) were mixed and layered onto a pre-coated slide with thin layers of 1% normal melting agarose in distilled water, and left for 30 min at 4 °C in order to allow the agarose to solidify. The slides were immersed in cold lysing solution overnight at 4 °C. The unit was filled with freshly prepared alkaline buffer (pH > 13). They remained submerged in the buffer for 20 min. Electrophoresis was carried out in the same buffer for 30 min at 25 V and 30 mA. Slides were then washed gently 2–3 times, at intervals of 5 min each with 0.4 M Tris pH 7.5. Then, the neutral buffer was drained, and each slide was stained with 50 μl of 20 μg/ml EtBr. The slides were visualized using × 40 objective of Leica epifluorescent microscope. The images for the cell nuclei were digitalized with truechrome retina screen camera version 4.2 build 5001. At least 100 cell nuclei for each sample were measured using the image analysis software (TnTekCometScoreTM freeware v1.5.) to obtain percentage of DNA in the head, percentage of DNA in the tail, tail length, tail moment, and olive moment.

### Karyotyping

Banding study was done according to [21, 22]. It occurs in four steps including culturing, harvesting, banding, and staining. Briefly, about half ml drops of blood were added to culture tube with the appropriate media to initiate mitosis. Afterward, two tubes were incubated at 37 ^°^C for 72 h. After stopping mitosis, the cell sediment was suspended by fixative solution. Cells were spread and dried on slides. The slides were treated by trypsin, stained by Giemsa solution (5%), and left to dry. The slides were examined by the Ziess microscope with automated stage coupled to metasystem image analyzer for chromosomal analysis. Twenty metaphases were analyzed for each case. Individual chromosomes were identified, arranged, and karyotyped according to ISCN, 2016.

### Statistical analysis

The data were statistically analyzed using the Statistical Package for Social Science (SPSS 20). For quantitative analysis, mean and standard deviation were calculated. Fisher’s exact test was used to examine the relationship between two qualitative variables. Student’s *t* test and paired *t* test were used to assess the statistical significance of the difference between two study group means and between two means measured twice for the same study group, respectively. ROC curve was used to determine the cutoff point in which highest sensitivity and specificity of several parameter as predictor for mortality.

## Results

The demographic analysis revealed that the mean age of the studied patients was 33.0 ± 11.7 years ranging from 18–55 years, with the majority of female cases (53.3%), while males represented (46.7%). Majority of patients were intoxicated due to suicidal attempts (76.7%), while 23.3% were exposed accidently. The GC analysis of the OP compounds showed that malathion was the most common type (40%) in the studied cases, followed by diazinon (30%) and chloropyrifos (30%). The delay time of studied patients ranged between 1 and 6 h with mean of 2.6 ± 1.1 h, while the mean duration of hospital stay was 5 ± 3.2 days ranging from 2 to 14 days. Mechanical ventilation was needed in 43% (*N* = 13) of our cases. The number of patients who survived was (*N* = 21, 70%) and 9 patients deceased (30%). Fisher’s exact test provided significant correlation between types of OP compound and mortality (*p* ≤ 0.05). Chloropyrifos represented the highest percentage among morbidity group (100%), followed by diazinon (66.7%) and malathion (50%). Moreover, the need for mechanical ventilation showed significant correlation with mortality (*N* = 9, *p* < 0.001), and the hospital stay duration with the mean of 5 days was also significantly correlated with mortality (*p* = 0.018).

Knowing that the mechanism of OP toxicity is via inhibition of AChE activity, the p.ChE activity was measured. The p.ChE activity was significantly decreased (*p <* 0.001) in OP patients before treatment, while it is significantly increased (*p <* 0.001) after treatment. Since OP acute toxicity has been reported to be through disruption of apoptosis and oxidative balance, we detected the biomarkers of these processes (Table 1). Concerning the oxidative stress biomarkers, serum TAC and MDA levels were highly significantly increased in OP poisoning cases before and after treatment compared with the control group (*p <* 0.001). For the apoptotic biomarkers, there was highly significant increase in the values of caspase 3 and 9 activities (*p <* 0.001) in OP cases before and after treatment when compared with the control group. Moreover, when comparing, caspase 3, caspase 9, MDA, and TAC levels in OP cases before and after treatment by using paired *t* test were significantly decreased (*p <* 0.001) after treatment. Our results showed the significant prognostic values of the selected biomarkers to be used as a tool for monitoring the effectiveness of therapy.

**Table 1:** Comparison between caspase 3, caspase 9, MAD, and TAC in acute OP poisoning cases before and after treatment (paired *t* test) and both compared to the control group (*t* test)

	Control	Cases before treatment		Cases after treatment		Paired ***t*** test
	Mean ± SD	Mean ± SD	*P* value	Mean ± SD	*P* value	*P* value
**Caspase 3 (ng/ml)**	0.37 ± 0.1	1.77 ± 0.46	< 0.001	1.08 ± 0.3	< 0.001	< 0.001
**Caspase 9 (ng/ml)**	0.85 ± 0.12	3.92 ± 1	< 0.001	2.44 ± 0.93	< 0.001	< 0.001
**MAD (nmol/ml)**	55.34 ± 7.87	264.8 ± 97.03	< 0.001	137.67 ± 44.94	< 0.001	< 0.001
**TAC (mM)**	0.32 ± 0.09	1.71 ± 0.29	< 0.001	0.99 ± 0.17	< 0.001	< 0.001
**p.ChE** (**U/l**)	6708.3 ± 653.11	966.23 ± 334.06	< 0.001	4110.13 ± 1775.74	< 0.001	< 0.001

*SD* standard deviation

*P* > 0.05: non-significant, *P* < 0.05: significant, *P* < 0.01: highly significant

**Fig. 1:**
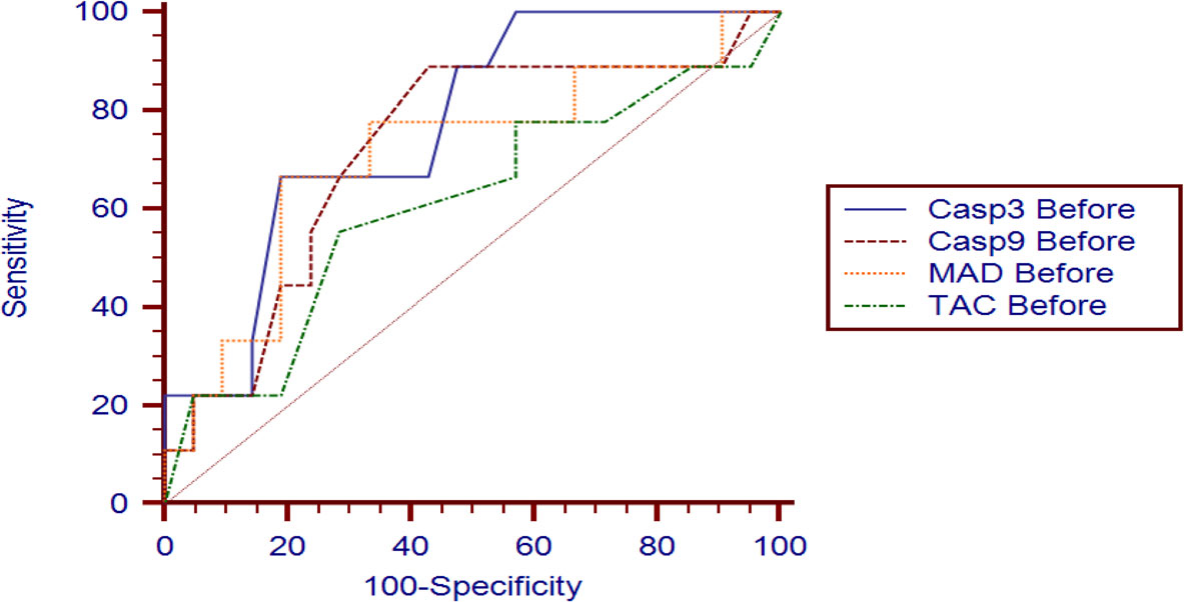
The receiving operating characteristic (ROC) curve for caspase 3, caspase 9, MAD and TAC in acute OP poisoning cases

To choose the most beneficial test to achieve our aim, the area under the ROC curve (AUC) was drawn to define the cutoff values of the selected biomarkers (Table 2, Fig. 1). We have used the data collected from the patients at the time of admission before treatment. The caspase 3 activity > 1.95 ng/ml was the best threshold to predict mortality. The caspase 3 activity of > 1.95 ng/ml showed AUC of 0.76 with positive predictive value of 60, sensitivity of 66.67 %, and specificity of 80.95%. In addition, caspase 9 cutoff of > 3.8 ng/ml showed the largest AUC 0.72 with positive predictive value of 47.1, sensitivity of 88.89%, and specificity of 57.14%. Mortality was 77% (7/9) and 88% (8/9) in patients with caspase 3 and 9 > 1.95 and > 3.8 ng/ml, respectively. Thus, caspases activity were significantly associated with increased mortality. Moreover, the need for invasive ventilation was more in patients with caspase 9 > 3.8 (10/13; 76.9%) rather than with caspase 3 > 1.95 (7/13; 53.8%). In addition, of the > 5-day hospital stay cases (*N* = 12), there were 4 and 9 cases higher than the cutoff values of caspase 3 and 9, respectively. The previous results indicate the importance of usage of the caspases as predictor for the patient’s outcomes. Further, in contrast to MDA and TAC ROC curve values (Table 2, Fig. 1), caspases better identified the patient who would die and showed more reliable mortality prediction than oxidative stress biomarkers for OP poisoning cases.

**Table 2:** Comparison between caspase 3, caspase 9, MAD, and TAC in mortality prediction for OP poisoning cases

Baseline	AUC	95% CI	***P*** value	Cutoff point	Sensitivity	Specificity	PPV	NPV
**Caspase 3**	0.767	0.578 to 0.901	0.0032	> 1.95	66.67	80.95	60	85
**Caspase 9**	0.72	0.527 to 0.867	0.0439	> 3.8	88.89	57.14	47.1	92.3
**MAD**	0.709	0.515 to 0.859	0.0682	> 310	66.67	80.95	60	85
**TAC**	0.608	0.414 to 0.780	0.3791	> 1.8	55.56	71.43	45.5	78.9

*PPV* positive predictive value, *NPV* negative predictive value, *(AUC)* area under curve

*P* > 0.05: non-significant, *P* < 0.05: significant, *P* < 0s.01: highly significant

**Fig. 2:**
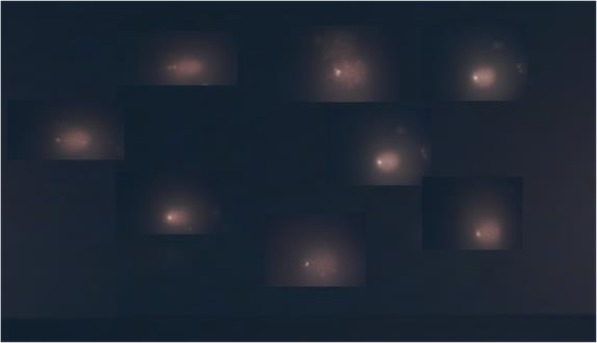
Comet assay of OP poisoning cases before treatment showing maximum damage

**Fig. 3:**
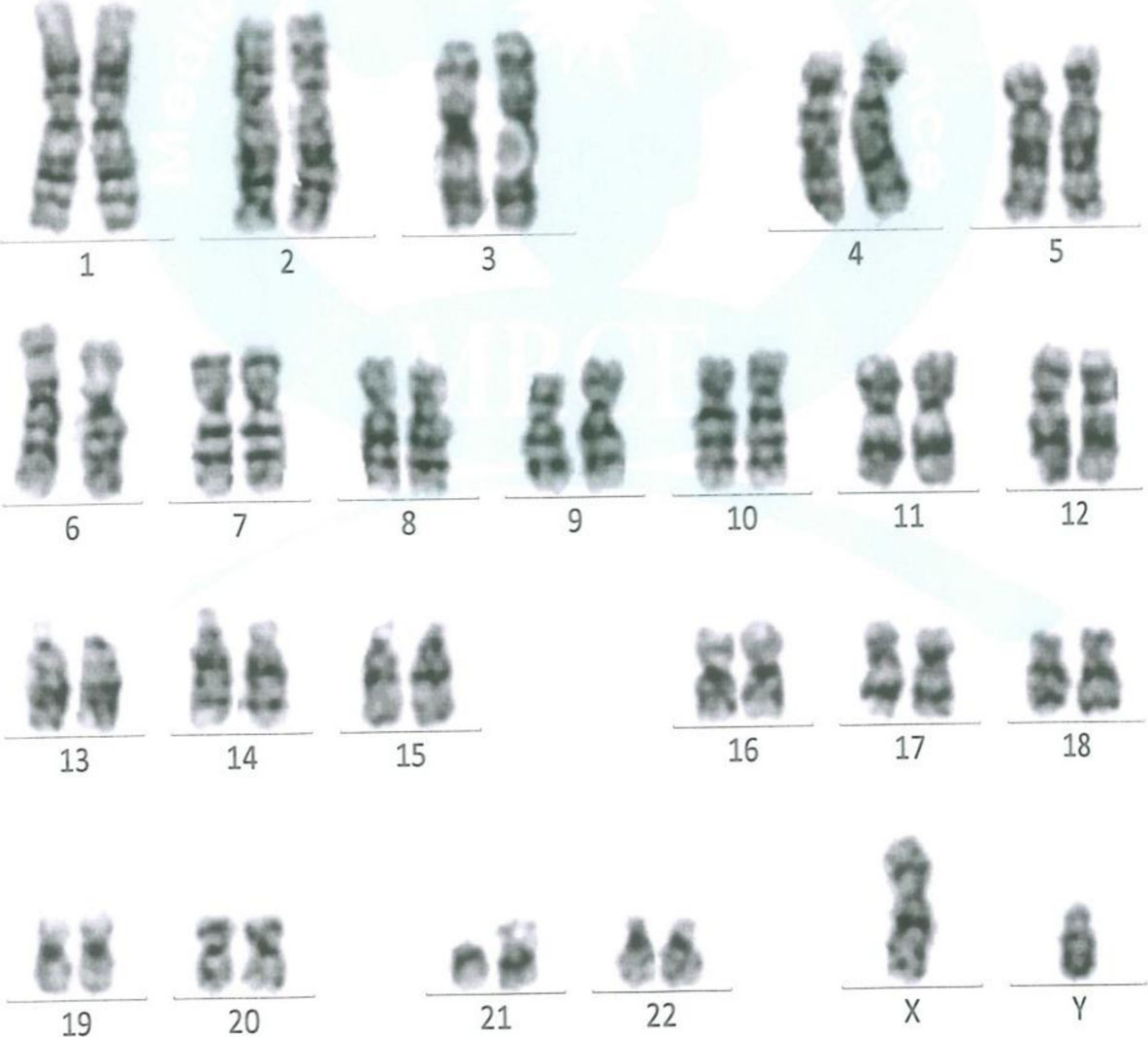
Karyotyping for male OP-intoxicated patient showing normal male karyotyping 46, XY: by G-banding technique

The apoptotic capacity of OP poisoning was also evaluated by examination of the genotoxicity via comet assay and chromosomal abnormalities by karyotyping. The comet assay results revealed that tail length and tail moment in OP poisoning cases significantly increased (*P <* 0.001) compared to control group (Table 3, Fig. 2). However, the results of the karyotyping revealed normal chromosomal pattern for both male and female OP poisoning cases (Fig. 3).

**Table 3:** Comparison of degree of DNA damage in OP poisoning cases

	Control		Cases		***t*** test
	Mean	SD	Mean	SD	*p* value
**%DNA in the head**	87.57	1.90	37.70	17.80	< 0.001
**Tail length (px)**	41.89	7.63	476.37	214.47	< 0.001
**%DNA in the tail**	12.07	1.71	62.30	17.80	< 0.001
**Olive moment**	11.93	2.61	82.19	14.45	< 0.001
**Tail moment**	7.52	2.80	362.23	203.16	0.001

*SD* standard deviation

*P >* 0.05: non-significant, *P* < 0.05: significant, *P* < 0.01: highly significant

## Discussion

OP compounds are the most common pesticides worldwide. Toxicity from these compounds results in fatal manifestation and deaths. Diagnosis of toxicity depends mainly on history of exposure, characteristic signs and symptoms of toxicity, and low serum cholinesterase levels [23]. In OP poisoning, inhibition of p.ChE activity can be used for diagnosis, but it is still controversy to be a prognostic marker. We sought that the need for prognostic and mortality predictive markers is crucial. Here, we could prove that caspases are significant prognostic biomarkers and mortality predictors for OP acute poisoning cases.

The current study revealed that the female patients were more than males in our study. The emotional liability of females to life stresses in addition to the easy availability of insecticides at home may be a reason for this observation [24]. On the other hand, Ahmed et al**.** suggested that males were more affected than females [25], due to the high unemployment rate, low income, and depression [26, 27]. The low cost and uncontrolled usage of these insecticides at homes, in addition to unstable emotional relationships and psychiatric disorders, may explain our results and others of the increased rate of the suicidal poisoning among cases [8, 25, 28]. Although our results and other’s [23] showed malathion as the most common among cases, other groups [29, 30] stated different percentages. This might reveal that the most common OP toxicity varies between the different environments and locations.

We went forward to our aim finding the most appropriate prognostic biochemical markers between the selected ones. In addition to the inhibition of p.ChE activity as a known marker in the OP poisoning cases, which is recovered after treatment [14, 31], we measured the other biomarkers of the oxidative stress and apoptosis. In line with Hundekari et al., our results showed MDA was significantly increased in OP poisoning cases and decreased after treatment [32]. This may be due to the toxic effects of OP compounds resulting in increasing the generation of ROS, which induces oxidative process, LPO damage of cell membranes, and high MDA level [33]. Our results agree with Ahmadi et al. about the increase of TAC in OP poisoning cases [11], which was explained by the adaptive response to the generated free radicals [34], and then we showed it decreased after treatment. Pesticides enhance generation of ROS and/or altered capacity of the antioxidant system of the body such as catalase (CAT), superoxide dismutase (SOD), and glutathione (GSH). This was also reflected by the increase in protein carbonyl contents as an excellent marker of protein oxidation and LPO [35, 36]. On the other hand, Ali et al. found significant decrease in serum TAC and GSH in dimethoate-treated rats [11]. Hundekari et al. also reported a significant decrease in plasma TAC in his study groups and a significant increase after treatment with atropine and pralidoxime. This may be explained by the overproduction of ROS, which causes significant LPO and consumption of antioxidant agents for which the body could not compensate in a short period [32]. Regarding the apoptotic markers, the present study showed that the activities of caspase 3 and 9 were significantly higher in cases before treatment, and these values decreased after treatment. The increase in activity of caspases after exposure to OP compounds may be due to OP-induced oxidative stress through releasing of cytochrome C that activates them to induce apoptosis [28]. Zhang et al. revealed**,** in accordance with our results, that caspase 9 and 3 activities increased in a concentration-dependent manner after OP exposure [37]. Also, Shiri et al. reported that the activities of caspases were increased in the diazinon-treated group [38]. OP-induced apoptosis may be induced either through extrinsic pathways (death receptors) or intrinsic pathways (mitochondria, DNA damage, and/or endoplasmic reticulum stress) and both pathways cause caspases activation [39].

In acute ChEI insecticides poisoning, low serum cholinesterase level supports the diagnosis of poisoning, but its significance in evaluation of treatment and mortality has been controversial [13, 14]. A new reliable biomarker is needed for the assessment of severity and evolution of patients with acute OP poisoning and prediction of the outcomes. Many biochemical markers have been used for the prediction of mortality in OP poisoning cases such as copeptin and creatine phosphokinase (CPK), C-reactive protein (CRP), glycemic status, and p.ChE [40, 41]. However, we sought that, since apoptosis and redox balance are the main processes involved in the OP acute poisoning, further study should be done on new biochemical markers for prediction of the outcomes of OP-intoxicated patients based on the biomarkers of those processes. Deaths were reported among 30% of the OP poisoning cases. Serum caspase 3 and 9 activities were positively and significantly associated with the mortality prediction, with a cutoff level of more than 1.95 and 3.8 ng/ml, respectively. Despite insignificant, we found that more than half of the mechanically ventilated cases had elevated levels of caspases above the cutoff values and more than half of the cases, who were hospitalized for >5 days, had elevated caspase 9 level above the cutoff value. These results may give powerful prove for the indicative values of caspases for the prediction of poor outcomes of OP poisoning.

Regarding the DNA fragmentation in OP poisoning cases, it significantly increased (*p* < 0.001). This could be attributed to DNA damage and apoptosis by the induction of the oxidative stress resulting from the pesticide [42]. D’Costa et al. are in accordance with the present study that observed significant DNA damage in all the fish treated with OP compounds [43]. Here, there was no effect of OP compound on chromosomal pattern as cases showed normal karyotyping. This could be attributed to the cytotoxic effect of OP compound, which masks the chromosomal aberrations. In addition, it can be explained by the decreased percentage of metaphase cells with structural aberrations depending on the OP compound dose [28]. Edwards and Tchounwou also reported no significant differences in aneuploidy, chromatid, and chromosome aberrations in OP-intoxicated patients [44–46]. On the other hand, Akyil et al. and Kapka-Skrzypczak et al. found that some OP compounds cause chromosomal aberrations. This chromosomal abnormality could be attributed to using various OP concentrations at different time intervals or by oxidative stress, which leads to DNA single-stranded breaks (SSB) as a result of the ROS production above the level of detoxification and repair [27, 47–49]. Moreover**,** the decrease in ROS scavenger and antioxidant enzymes causes nucleic acid, protein, and lipid damage, leading to induction of chromosomal instability and mutations [22]. We here recommend further prospective study involving larger sample size and studied cases with known OP compound concentrations should be conducted to verify the effect of OP poisoning.

## Conclusions

We found that serum caspases activity are of a significant role in the evaluation of acute OP poisoning treatment, as well as a better mortality predictive role in OP poisoning cases. We suggest measuring serum caspases activity at time of admission to be used for prognostication in acute OP poisoning cases that could help in identifying patients in need of intensive monitoring and treatment. We also recommend the anti-apoptotic and anti-oxidant therapeutic as future strategy to decrease the OP acute poisoning severity.

## Data Availability

All data generated or analyzed during this study are included in this manuscript.

## Abbreviations

AChE: Acetyl cholinesterase
AUC: Area under curve
CAT: Catalase
ChEI: Cholinesterase inhibitor
CPK: Creatine phosphokinase
CRP: C-reactive protein
DNA: Deoxyribonucleic acid
ELISA: Enzyme linked immunosorbent assay
GSH: Glutathione
LPO: Lipid peroxidation
MDA: **M**alondialdehyde
NPV: Negative predictive value
OP: Organophosphorus
PPV: Positive predictive value
p.CHE: Pseudo-cholinesterase
ROC: Receiver operating characteristic
ROS: Reactive oxygen species
SPSS: Statistical Package for Social Science
SCGE: Single cell gel electrophoresis
SSB: Single-stranded breaks
SOD: Superoxide dismutase
TAC: Total antioxidant capacity
WHO: World Health Organization

## References

[1] Viswanathan KG, Gupta A, Santhosh CS (2014). Profile of fatal organophosphate pesticide poisoning cases near Davangere. J Punjab Acad. Forensic Med. and Toxicol.

[2] Tripathi S (2014). Prognostic value of Glasgow Coma Scale, Poisoning Severity Score and serum acetylcholinesterase levels in organophosphorus poisoning. J Evolution Med Dental Sci..

[3] Poisoning Control Center-Ain shams University. (2017) Annual Report of Poisoning Control Center (unpublished).

[4] Jokanović M, Prostran M (2009). Pyridinium oximes as cholinesterase reactivators. Structure-activity relationship and efficacy in the treatment of poisoning with organophosphorus compounds, Current Medicinal Chemistry.

[5] Tunsaringkarn T, Zapuang K, Rungsiyothin A (2013). The correlative study of serum pseudo-cholinesterase, biological parameters and symptoms among occupational workers. Indian J Clin Biochem..

[6] Mood M, Saber H (2012). Recent advances in the treatment of organophosphorous poisonings. Iranian Journal of Medical Sciences.

[7] Gupta PK, GUPTA RC (2007). Toxicity of herbicides. Veterinary toxicology: basic and clinic principles.

[8] Kumar E (2012). Current review on organophosphorus poisoning. Scholars Research Library.

[9] Mishra BP, Badade ZG, Rastogi SK (2013). Antioxidant status and oxidative stress in organophosphate pesticide poisoning. Journal of Dental and Medical Sciences.

[10] Ahmadi N, Mandegary A, Jamshidzadeh A (2018). Hematological abnormality, oxidative stress, and genotoxicity induction in the greenhouse pesticide sprayers; investigating the role of NQO1 gene polymorphis. Toxics.

[11] Ali MWM, Gomaa MMS, Shalby SMS, et al. (2017) Study of chronic toxic effect of deltamethrin and di methoate on brain of adult male albino rat. Zagazig J. Forensic Med. & Toxicol 15 No. (1).

[12] Amanvermez R, Baydın A, Yardan T (2010). Emergency laboratory abnormalities in suicidal patients with acute organophosphate poisoning. Turkish Journal of Biochemistry.

[13] Bhattacharyya K, Phaujdar S, Sarkar R (2011). Serum creatine phosphokinase: a probable marker of severity in organophosphorus poisoning. Toxicol Int..

[14] Pradeepkumar H, Rangappa P, Jacob I (2016). Pseudocholinesterase as a predictor of mortality and morbidity in organophosphorus poisoning. Indian Journal of Critical Care Medicine.

[15] Karki P, Ansari JA, Bhandary S (2014). Cardiac and electrocardiographical manifestations of acute organophosphate poisoning. Singapore Med J.

[16] Wolf M, Deleu R and Copcn A. (2005) Separation of pesticides by capillary gas chromatography.Organophosphorus insecticides.journal of separation sciences 2 (4); (7).

[17] Young DS. (2000) Effect drugs on Clinical Lab. Test, 5^th^ Ed. AACC Press.

[18] Koracevic D, Koracevic G (2001). Method for the measurement of antioxidant activity in human fluids. J Clin Pathol.

[19] Satoh K (1978). Serum lipid peroxide in cerebrovascular disorders determined by a new colorimetric method Clinica Chimica Acta.

[20] Singh NP, Coy MC, Tice RR (1988). A simpletechnique for quantification of low levels of DNA damage in individual cells, Exp. Cell. Res.

[21] Seabright M. (1971) A rapid banding technique for human chromosomes. Lancet.II 971-972. 10.1016/s0140-6736(71)90287-x 4107917

[22] Verma RS, Babu A (1995). Human chromosomes: principles and techniques.

[23] Badiger S, Harish J (2017). Study the clinical and electrocardiographic changes in organophosphorus poisoning. Sch. J. App. Med. Sci..

[24] Li Q, Kobayashi M, Kawada T (2015). Carbamate pesticide-induced apoptosis in human T lymphocytes *Int*. J. Environ. Res. Public Health..

[25] Ayse FN, Omer A, lhan D. (2016). Protective effect of intravenous lipid emulsion treatment on malathioninduced ovarian toxicity in female rats. European Review for Medical and Pharmacological Sciences.

[26] Savu O, IonescuTirgoviste C, Atanasiu V (2012). Increaseintotalantioxidant capacity of plasma despite high levels of oxidative stress in uncomplicated type 2 diabetes mellitus. J.Int. Med. Res..

[27] Ramazani M, Qujeq D, Moazezi Z (2019). Assessing the levels of L-carnitine and total antioxidant capacity in adults with newly diagnosed and long-standing type 2 diabetes. Canadian Journal of Diabetes. Volume.

[28] Ahmed S, Das B, Nadeem A (2014). Survival pattern in patients with acute organophosphate poisoning on mechanical ventilation: a retrospective intensive care unit-based study in a tertiary care teaching hospital. Indian Journal of Anaesthesia.

[29] Abd El AL AA, Fawzi MM, ALKhafif MA, et al. (2016) Epidemiological study of organophosphorus compounds insecticides types related to acutely intoxicated patients presented to Poison Control Center (PCC-ASU)–Egypt. IOSR Journal of Environmental Science, Toxicology and Food Technology 10(6): PP 72-78

[30] Kumar A, Virupakshappa V (2017). Creatinephosphokinase in organophosphorus poisoning. Int J Adv Med.

[31] Badiger S, Vishok M (2016). Study of serum amylase and serum cholinesterase in organophosphorus poisoning. Journal of Krishna Institute of Medical Sciences University.

[32] Hundekari AI, Suryakar AN, Rathi BD (2016). Oxidative stress and antioxidant status in acute organophosphorous pesticides poisoning cases of North Karnataka (India). Journal of Environmental Health Research.

[33] Pore N, Pujari K, Jadkar S (2018). Oxidative stress markers in patients with organophosphorus poisoning. International Journal of Biotechnology and Biochemistry.

[34] Sargazi Z, Nikravesh MR, Jalali M (2016). Apoptotic effect of organophosphorus insecticide diazinon on rat ovary and protective effect of vitamin E. Iranian Journal of Toxicology.

[35] Androutsopoulos VP, Hernandez AF, Liesivuori J (2013). Mechanistic overview of health associated effects of low levels of organochlorine and organophosphorous pesticides. Toxicology.

[36] Dardiotisa E, Aloizoua A, Siokasa V (2019). Paraoxonase-1 genetic polymorphisms in organophosphate metabolism. Toxicology.

[37] Zhang Y, Chang Y, Cao H (2018). Potential threat of chlorpyrifos to human liver cells via the caspase-dependent mitochondrial pathways. Food and agriculture immunology.

[38] Shiri M, Navaei-Nigjeh M, Baeeri M (2016). Blockage of both the extrinsic and intrinsic pathways of diazinon-induced apoptosis in PaTu cells by magnesium oxide and selenium nanoparticles. International Journal of Nanomedicine.

[39] Kankaya E, Kaptaner B (2014). Increased apoptosis in the liver of chalcalburnus tarichi exposed to sublethal concentrations of methyl parathion. Journal of Applied Biological Sciences.

[40] Sumathi ME, Harish Kumar S, Shashidhar KN et al (2014) Prognostic significance of various biochemical parameters in acute organophosphorus poisoning. Toxicol Int. 21(2):167–171 10.4103/0971-6580.139800 PMC4170558 25253926

[41] Wu X, Xie W, Cheng Y (2016). Severity and prognosis of acute organophosphorus pesticide poisoning are indicated by C reactive protein and copeptin levels and APACHE 2 score. J Exp Therap Med..

[42] Jacobsen-Pereira CH, dosSantos CR, Maraslis FT (2018). Markers of genotoxicity and oxidative stress in farmers exposed to pesticides. Ecotoxicol. Environ. Saf..

[43] D’Costa AH, Shyama SK, Praveen Kumar MK (2018). Induction of DNA damage in the peripheral blood of zebrafish (Danio rerio) by an agricultural organophosphate pesticide, monocrotophos. Int Aquat Res..

[44] Edwards FL, Tchounwou BP (2005). Environmental toxicology and health effects associated with methyl parathion exposure – a scientific review. Int. J. Environ. Res. Public Health.

[45] Alfonso M, Durán R, Fajardo D (2019). Mechanisms of action of paraoxon, an organophosphorus pesticide, on in vivo dopamine release in conscious and freely moving rats. Neurochemistry International..

[46] Yahia D, Marwa F (2019). Cytogenetic and genotoxic effects of penconazole and chlorpyrifos pesticides in bone marrow of rats. J. Advanced Veterinary Res.

[47] Akyil D, Konuk M, Eren Y (2017). Mutagenic and genotoxic effects of Anilofos with micronucleus, chromosome aberrations, sister chromatid exchanges and Ames test. Cytotechnology.

[48] Kapka-Skrzypczak L, Cyranka M, Skrzypczak M et al (2011) Biomonitoring and biomarkers of organophosphate pesticides exposure – state of the art. Annals of Agricultural and Environmental Medicine 18(2) 22216802

[49] Nwani CD, Nagpure NS, Kumar R (2011). Mutagenic and genotoxic assessment of atrazinebased herbicide to freshwater fish Channa punctatus (Bloch) using micronucleus test and single cell gel electrophoresis. Environ Toxicol Pharmacol.

